# Recent advances in deciphering oligodendrocyte heterogeneity with single-cell transcriptomics

**DOI:** 10.3389/fncel.2022.1025012

**Published:** 2022-10-13

**Authors:** Lukas Valihrach, Zuzana Matusova, Daniel Zucha, Ruslan Klassen, Sarka Benesova, Pavel Abaffy, Mikael Kubista, Miroslava Anderova

**Affiliations:** ^1^Laboratory of Gene Expression, Institute of Biotechnology of the Czech Academy of Sciences, Vestec, Czechia; ^2^Department of Cellular Neurophysiology, Institute of Experimental Medicine of the Czech Academy of Sciences, Prague, Czechia; ^3^Faculty of Science, Charles University, Prague, Czechia; ^4^Department of Informatics and Chemistry, Faculty of Chemical Technology, University of Chemistry and Technology, Prague, Czechia; ^5^Department of Biochemistry and Microbiology, Faculty of Food and Biochemical Technology, University of Chemistry and Technology, Prague, Czechia; ^6^TATAA Biocenter AB, Gothenburg, Sweden

**Keywords:** oligodendrocyte, heterogeneity, scRNA-seq, snRNA-seq, populations, marker genes

## Abstract

Oligodendrocytes (OL) have been for decades considered a passive, homogenous population of cells that provide support to neurons, and show a limited response to pathological stimuli. This view has been dramatically changed by the introduction of powerful transcriptomic methods that have uncovered a broad spectrum of OL populations that co-exist within the healthy central nervous system (CNS) and also across a variety of diseases. Specifically, single-cell and single-nucleus RNA-sequencing (scRNA-seq, snRNA-seq) have been used to reveal OL variations in maturation, myelination and immune status. The newly discovered immunomodulatory role suggests that OL may serve as targets for future therapies. In this review, we summarize the current understanding of OL heterogeneity in mammalian CNS as revealed by scRNA-seq and snRNA-seq. We provide a list of key studies that identify consensus marker genes defining the currently known OL populations. This resource can be used to standardize analysis of OL related datasets and improve their interpretation, ultimately leading to a better understanding of OL functions in health and disease.

## Introduction

OLs represent a type of glial cells found in the CNS of invertebrates and vertebrates. Their primary role is to envelop the axons of the neurons in myelin, which provides insulation and maintains the electrical impulse conduction. Since their first description in 1921, they have been considered a heterogeneous population, displaying variable morphology and spatial distribution (Del Rio-Hortega, 1921). However, decades of subsequent research have led to the general understanding that OLs are instead a homogenous population of cells, without any major functional heterogeneity. It was not until recently that advances in single-cell analysis revealed a new spectrum and sources of OL heterogeneity, including their variations related to differentiation state, developmental origin, anatomical site, age and sex (for an extensive review, see Seeker and Williams, 2022).

A completely new area of OL characterization started with the advent of single-cell transcriptomic techniques allowing analysis of thousands of cells, each characterized by the activity of thousands of genes. The first landmark studies characterizing OL transcriptional heterogeneity were performed on single cells in healthy animals (Zeisel et al., 2015, 2018; Marques et al., 2016). The introduction of protocols for the analysis of single nuclei isolated from archived samples facilitated the expansion and application of the technique to investigations from human tissues. Currently, we are experiencing a boom in transcriptional studies characterizing OLs in a variety of pathological conditions and disease states, rapidly extending our understanding of OL heterogeneity. However, with the increasing number of scRNA-seq and snRNA-seq studies, and the enormous complexity of the information embedded within each dataset, there is a new challenge for OL researchers that is the comparison and the interpretation of newly acquired data with existing studies. Although this step is not mandatory and often missed in reports, it provides an important insight into the general function of OLs, potentially transferring knowledge derived from one particular model to a broader spectrum of pathological states.

The process of data interpretation using other studies as reference is typically done by comparing selected marker genes to a defined OL population or by integrative analysis. The relation of OL populations is then assessed by the overlap of these marker genes or by enrichment type of analysis. The downside is that the calculation of marker genes is heavily influenced by the data processing and the particular downstream analysis, which biases the comparison of populations. Integrative analysis is therefore a more robust way to interpret new data (Stuart and Satija, 2019). Integration allows merging of data for the unified processing of multiple datasets, even if they are derived using different protocols or experimental models. Although more robust, this method is biased by the choice and settings of the integration tool. Finally, the choice of reference studies is of uttermost importance. This is far from trivial as features of OL heterogeneity of interest, may be hidden in studies characterizing completely unrelated biological questions.

To guide OL researchers in the wealth of current knowledge, we prepared a compact review summarizing the current understanding of OL heterogeneity in health and disease based on single-cell and single-nucleus transcriptomic technologies. Our motivation is to provide the community a unified overview of key transcriptomic studies dealing with OL heterogeneity in the mammalian CNS (Table 1) and consensus marker genes of selected OL populations (Table 2). We hope that the interpretation of new datasets with respect to those already available will lead to a standardization of OL nomenclature and our better understanding of their transcriptional heterogeneity.

**TABLE 1:** Selection of key transcriptomic studies for understanding OL heterogeneity in health and disease*.

Study	Species (mouse/human)	Condition	Methods	# of OL cluster (incl. OPCs)	Significance	Accession number (custom database if available)
Zeisel et al. (2015)	Mouse	Ctrl	scRNA-seq	6	First large-scale scRNA-seq study of all cell types in mouse CTX and HC	GSE60361 (link)
Marques et al. (2016)	Mouse	Ctrl	scRNA-seq	12	First large-scale OL focused scRNA-seq study in mouse CNS	GSE75330 (link)
Zeisel et al. (2018)	Mouse	Ctrl	scRNA-seq	10	First large-scale scRNA-seq of all cell types in mouse CNS	SRP135960 (link)
Falcao et al. (2018)	Mouse	Ctrl, EAE	scRNA-seq	14	First scRNA-seq defining disease related OL clusters	GSE113973 (link)
Jakel et al. (2019)	Human	Ctrl, MS	snRNA-seq	9	First OL focused snRNA-seq of MS patients and healthy controls	GSE118257 (link)
Mathys et al. (2019)	Human	Ctrl, AD	snRNA-seq	5	First large-scale snRNA-seq of AD patients and healthy controls	syn18485175
Floriddia et al. (2020)	Mouse	Ctrl, SCI	ISH/ISS, scRNA-seq	11	Spatial distribution of mature OLs in WM and GM of murine brain and SC	GSE128525 (link)
Zhou et al. (2020)	Both	Ctrl, AD	snRNA-seq	2–5	Description of *Serpina3n*^+^ *C4b*^+^ reactive OLs in AD mice	GSE140511, syn21125841
Chen et al. (2020)	Both	Ctrl, AD	ISS, ST	–	Spatial characterization of plaque-induced transcriptomic response in AD	GSE152506, syn22153884 (link)
Lee et al. (2021)	Mouse	Ctrl, AD	scRNA-seq	7–8	Description of two disease-associated OL clusters across three AD models	GSE160512, GSE181786, GSE153895
Yao et al. (2021)	Mouse	Ctrl	sc + snRNA-seq, snATAC-seq, snmC-seq2	9	BICCN—transcriptomic and epigenomic cell atlas of mouse CTX	nemo:dat-ch1nqb7 (link)
Bakken et al. (2021)	Both	Ctrl	snRNA-seq, snmC-seq2, SNARE-seq2	4–9	BICCN—comparison of motor CTX in human, marmoset and mouse	nemo:dat-ek5dbmu (link)
Russ et al. (2021)	Mouse	Ctrl	sc + snRNA-seq	5	Harmonized atlas of mouse SC cell types (six integrated datasets)	GSE158380 (link)
Morabito et al. (2021)	Human	Ctrl, AD	snRNA-seq, snATAC-seq	14–15	Chromatin accessibility and transcriptomic characterization of AD	syn22079621 (link)
Bartosovic et al. (2021)	Mouse	Ctrl	scCUT&Tag	5	scCUT&Tag profiling of histone modification and TFs in murine OLs	GSE163532 (link)
Hilscher et al. (2022)	Mouse	Ctrl	ISS	12	Spatial distribution of OL populations from Marques et al. (2016)	Data not available
Sadick et al. (2022)	Human	Ctrl, AD	snRNA-seq	7	OL integration in multiple human AD datasets	GSE167494 (link)
Kenigsbuch et al. (2022)	Mouse	Ctrl, EAE, aging	scRNA-seq	14	Definition of DOLs in murine models of AD, MS and aging	GSE202297
Kaya et al. (2022)	Mouse	Aging	scRNA-seq	4–7	Identification of interferon-responsive OLs during WM aging	Data not yet released
Yadav et al. (2022)	Human	Ctrl	snRNA-seq, ST	8	Cellular taxonomy of adult human SC	GSE190442 (link)
Meijer et al. (2022)	Both	Ctrl, EAE	snATAC-seq, multiome	12	In-depth epigenomic analysis of immune genes in OLs	GSE166179 (link)
Pandey et al. (2022)	Both	AD, MS	sc + snRNA-seq, smFISH	4–17	Characterization of three OL activation states across disease models	GSE180041, GSE182846 (link)

*For a curated database of all available single-cell transcriptomics studies with key experimental information (see Svensson et al., 2020).

Ctrl, healthy conditions; EAE, experimental autoimmune encephalomyelitis; MS, multiple sclerosis; AD, Alzheimer’s disease; SCI, spinal cord injury; ISH, *in situ* hybridization; ISS, *in situ* sequencing; snATAC-seq, single-nucleus assay for transposase-accessible chromatin using sequencing; snmC-seq2, single nucleus methylcytosine sequencing; SNARE–seq2, single-nucleus chromatin accessibility and messenger RNA expression sequencing; snRNA/ATAC multiome, Chromium Single Cell Multiome ATAC + Gene Expression; ST, Spatial transcriptomics (Visium, 10x Genomics); smFISH, multiplexed single-molecule fluorescence *in situ* hybridization; CTX, cortex; HC, hippocampus; WM/GM, white/gray matter; SC, spinal cord; TFs, transcription factors; DOLs, disease-associated oligodendrocytes.

**TABLE 2:** Marker genes of selected OL populations as reported by authors.

Study	OL clusters	Gene signature	Source
Marques et al. (2016)	OPC	*Ptprz1, Pdgfra, Serpine2, Cspg5, Vcan, Cspg4*	
			Supplementary Table 1—Top 6 marker genes of each branch of the dendrogram in **Figure 1C** (out of 50). For subclusters of the same differentiation stage, top 3 genes defining the stage and top3 genes defining the subcluster were selected. Of note, the list of genes is strictly related to the dendrogram in **Figure 1C**.
	COP	*Cd9, Neu, 43110035E14Rik, Bmp4, Gpr17, Vcan*	
	NFOL1	*9630013A20Rik, Arpc1b, Tmem2, Chn2, Mpzl1, Frmd4a*	
	NFOL2	*9630013A20Rik, Arpc1b, Tmem2, Mobp, Ddr1, Tspan2*	
	MFOL1	*Ctps, Tmem141, Opalin, 9630013A20Rik*	
	MFOL2	*Ctps, Tmem141, Opalin, Mal, Ptgds, Evi2a-evi2b*	
	MOL1	*Apod, Sepp1, S100b, Fosb, Dusp1, Dnajb1*	
	MOL2	*Apod, Sepp1, S100b, Anxa5, Klk6, Mgst3*	
	MOL3	*Apod, Sepp1, S100b, Car2, Cntn2, Gad2*	
	MOL4	*Apod, Sepp1, S100b, Serpinb1a, Neat1, Sepp1*	
	MOL5	*Apod, Sepp1, S100b, Cyp51, Dhcr24, Pdlim2*	
	MOL6	*Apod, Sepp1, S100b, Il33, Apoe, Ptgds*	
Zeisel et al. (2018)	OPC	*Pdgfra, C1ql1, Sapcd2, Emid1, Lhfpl3*	
			Supplementary Table 4—Combination of markers genes uniquely identifying populations based on “trinarization” scoring procedure developed by authors.
	COP1	*Neu4, Brca1, Bmp4, Pak4, Lims2*	
	COP2	*Tnr, Rinl, Gpr17, Enpp6, Pdcd4*	
	NFOL1	*Cnksr3, H2-Ab1, Rras2, Il23a, Tmem163*	
	NFOL2	*Tmem2, Gm26834, Rras2, Itpr2, Sema4d*	
	MFOL1	*Ccp110, Snx33, Hhip, Tmem88b, Arap2*	
	MFOL2	*2210011C24Rik, Wfdc18, Tmem141, Birc2, Fam214a*	
	MOL1	*Opalin, Ninj2, Efhd1, Mal, Ppp1r14a*	
	MOL2	*Hapln2, Dock5, Anln, Ugt8a, Gjb1*	
	MOL3	*Klk6, Nkx2-9, Cdkn1c, Rab37, 2700046A07Rik*	
Falcao et al. (2018)	EAE_m1	*Serpina3m, Klk8, Serpina3c, Serping1, Irf7, Cd74, Ifih1*	
			Supplementary Figure 4B—Representative genes for EAE-associated modules (sortable list in Supplementary Table 1)
	EAE_m3	*Lyz2, C4b, Serpina3n, Klk6, Igtp, Irgm2, Ccdc13*	
	EAE_m13	*Plin4, Hif3a, Fam107a, Phyhd1, Cdkn1a, Sult1a1*	
Jakel et al. (2019)	ImOLs	*ARHGAP24, MEF2C, C10orf11, APOE, CD74, DOCK8, PLXDC2, ELL2, APBB1IP, HLA.DRA, C3, PTPRC*	
			Supplementary Figure 8C—Selection of key markers (sortable list in Supplementary Table 4)
Zhou et al. (2020)	Reactive OLs	*C4b, Serpina3n, H2-d1*	
			**Figure 2A**—Three highlighted marker genes (sortable list in Supplementary Table 1)
Lee et al. (2021)	MOL-DA1	*C4b, Serpina3n*	
			**Figure 2D**—Selected marker genes (full list in Supplementary Table 8)
	MOL-DA2	*C4b, Serpina3n, Cdkn1a, Ddit3, Gadd45a*	
Yao et al. (2021)	OPC Pdgfra	*Col14a1, Cnr1, Gad2, Spock3, Sema3c, Zfp385b*	
			Supplementary Table 6—Top 6 marker genes for each consensus OL population (full list available)
	Oligo Enpp6_1	*Col14a1, Kcnip1, Cxcl14, Spock3, Sema3c, Chrna7*	
	Oligo Enpp6_2	*Col14a1, Gad2, Cxcl14, Cnr1, Rab3c, A830018L16Rik*	
	Oligo Enpp6_3	*Col14a1, Grik1, Cxcl14, Spock3, Sema3c, Necab1*	
	Oligo Enpp6_4	*Kcnip1, Gad1, Col14a1, Gad2, Grik1, Pax6*	
	Oligo Opalin_1	*Adarb2, Grip1, Col14a1, Kcnip1, Gad1, Dab1*	
	Oligo Opalin_2	*A830018L16Rik, Kcnip1, Col14a1, Grik1, Slc2a13, Gad1*	
	Oligo Opalin_3	*Grip1, Col14a1, Kcnip1, A830018L16Rik, Grik1, Gad1*	
	Oligo Opalin_4	*Col14a1, Kcnip1, Gad1, Maf, Shisa9, Neto1*	
Sadick et al. (2022)	Int0	*SVEP1, LINC01608, PLXDC2, DYSF*	
			**Figure 3E**—Selection of top markers of four integrated OL datasets (sortable list in Supplementary Table 5)
	Int1	*CTNNA2, CNDP1, ST3GAL6, QDPR, CRYAB*	
	Int2	*FP236383.3, MT-ND4, MT-ND3, MT-CO2, MT-ATP6*	
	Int3	*ACTN2, SLC5A11, RASGRF1, LINC00609, ANKRD18A*	
	Int4	*SGCZ, MDGA2, CNTN1, KCNIP4, FRY*	
	Int5	*RBFOX1, AFF3, ACSBG1, COL18A1*	
	Int6	*NRP2, LUCAT1, NAV2, CAMK2D, NEAT1*	
Kenigsbuch et al. (2022)	DOLs	*Serpina3n, C4b, H2-d1, H2-k1, B2m, Il33, Klk6, CD9, CD63*	**Figure 2F**—Highlighted marker genes
Pandey et al. (2022)	MOL-DA1	*Serpina3n, C4b, Anxa2, Plvap, Thbs3, Steap3, Emp3, Parvb, S100a10, Tnfrsf1a, Col6a1, Sema4f*	**Figure 2B**—Selected marker genes (sortable list in Supplementary Table 2)
	MOL-DA2	*Cdkn1a, Bax, Ddit3, Fos, Atf4, Egr1, Ccnd1, Tnfrsf12a, Btg1, Egr2, Klf4, Fgf7, Rrad, Gdf15*	
	MOL-INF	*H2-d1, Stat1, Bst2, Igtp, Psmb8, Irgm1, Ifit1, Irf7, Psme1, Oasl2, H2-q4, Tap1, Ifit2*	
Kaya et al. (2022)	AROs	*C4b, Serpina3n, Socs3, Vim, Gadd45a, Bbc3*	
			**Figure 1G**—Highlighted marker genes
	IROs	*Ifi27l2a, H2-k1, Usp18, B2m, Stat1*	

OPC, oligodendrocyte precursor cells; COP, committed OL; NFOL, newly formed OL; MFOL, myelin forming OL; MOL, mature OL; EAE, experimental autoimmune encephalomyelitis; ImOLs, immune OLs; MOL-DA, mature OL disease-associated; Int, integrated cluster of OLs, DOLs, disease-associated OLs; MOL-INF, mature OL interferon-associated; AROs, aging-related OLs; IROs, interferon-responsive OLs.

## Oligodendrocyte heterogeneity in health

Since the advent of scRNA-seq analysis, there have been efforts to classify the CNS cell types. Early datasets comprised only of tens of cell types represented by hundreds to thousands of cells. The low proportion of OLs did not allow for their in-depth characterization or the OLs were not the primary focus of the studies. The first milestone deciphering OL heterogeneity was made in murine CNS, in studies published by researchers from the Division of Molecular Neurobiology at Karolinska Institute (Zeisel et al., 2015, 2018; Marques et al., 2016).

The study of Zeisel et al. (2015) focused on somatosensory cortex and hippocampal CA1 region of juvenile mice, and analyzed over 3,000 cells, including more than 800 OLs. Clustering revealed six OL populations representing various stages of maturation: post-mitotic, immature, pre-myelinating, myelinating, intermediate, and terminally differentiated post-myelination OLs. Marques et al. (2016) provided further details by analyzing over 5,000 cells of OL lineage in 10 regions of murine juvenile and adult CNS. In total, they observed 12 clusters of OL lineage, representing a continuum from oligodendrocyte precursor cells (OPC) to mature OLs (OPC, COP, NFOL1-2, MFOL1-2, and MOL1-6; Table 2). Whereas the initial stages of OL maturation (to MFOL1-2) were found sequential and uniform across CNS regions, mature OLs showed regional specificity, being present in unique proportions in each brain region. Moreover, cells sampled from adult mice comprised mostly of OPCs and two populations of mature OLs (MOL5-6), while juvenile cells were represented by the full spectrum of the OL populations. Lastly, the study of Zeisel et al. (2018) expanded the scope of the previous datasets by analyzing 19 CNS regions, counting over half a million of cells, with a large fraction comprising of OLs. Their analysis identified 10 clusters of OL lineage (OPC, COP1-2, NFOL1-2, MFOL1-2, MOL1-3; Table 2), but even with the increased sample size, it did not reveal any additional OL subtypes beyond those already described by Marques et al. (2016). Notably, despite the large similarity of the two datasets, there was not a perfect cluster match, probably due to different scRNA-seq technology and data processing protocols used. These differences might be possible to remove with integrative analysis, standardizing the nomenclature, and defining a set of consensus marker genes for future studies. For now, the marker genes and the reference for interpretation of new datasets might be selected based on specific preferences. While the annotation used by Marques et al. (2016) has been applied in several subsequent studies (Falcao et al., 2018; Jakel et al., 2019; Floriddia et al., 2020; Bartosovic et al., 2021; Hilscher et al., 2022; Meijer et al., 2022; Pandey et al., 2022) and has become a standard in the field, an advantage of the Zeisel et al. (2018) annotation are the region-specific references rich in OLs, whose transcriptome was measured with the widely used 10x Genomics technology.

The classification of OLs in human CNS lagged behind the progress in mouse because of practical constrains in obtaining fresh samples for isolation of single cells. This has since changed with the introduction of protocols for the analysis of single nuclei, making it possible to process archived samples from the human brain biobanks. Using snRNA-seq, the first studies comprised of only a small number of OLs, limiting the annotation to a few clusters vaguely reflecting OL maturation (Habib et al., 2017; Lake et al., 2018). The first comprehensive characterization was a study describing altered OL heterogeneity in the white matter (WM) of five healthy donors and four individuals with progressive multiple sclerosis (MS) (Jakel et al., 2019). The authors identified seven clusters of mature OLs (Oligo1-6 and ImOLs), and clusters of OPC and committed oligodendrocyte precursors (COP). Integrative analysis with two previous datasets (Habib et al., 2017; Lake et al., 2018), re-annotated the Oligo6 cluster to an intermediate state, connecting the OPC and COP clusters with the mature OLs. Immune OLs (ImOLs) resembled the OPC and COP, but also expressed immune response related genes (Table 2). Comparison of the human clusters with those previously obtained for the mouse OLs by Falcao et al. (2018), revealed similarities between the two species. In short, several other publications characterizing OL heterogeneity in CNS diseases have appeared (see next chapter). These reported varying numbers of clusters, most likely affected by the particular experimental design and data-processing pipeline used. It is likely that the complexity of human CNS will require dedicated efforts to determine and annotate the full spectrum of human OL heterogeneity. The first step in this direction was recently taken by Sadick et al. (2022), who integrated their data with three other studies (Grubman et al., 2019; Mathys et al., 2019; Zhou et al., 2020) identifying seven OL populations (Table 2), that appeared consistently across the datasets. However, an in-depth functional characterization was not performed. Of note, a similar integrative approach was used to create a harmonized atlas of mouse spinal cord cell types, but with limited details of OLs (Russ et al., 2021). Lastly, a comprehensive cellular taxonomy of the adult human spinal cord was recently released by Yadav et al. (2022), including an integrative analysis of mouse and human data.

Leaving the strictly OL-oriented research, the BRAIN Initiative Cell Census Consortium (BICCC) provides a great source of information that reveals additional layers of OL heterogeneity. Recently, BICCN released results of its first implementation phase presenting a multimodal cell census and an atlas of the mammalian primary motor cortex (BICCN, 2021). This massive resource provides a detailed transcriptomic and epigenomic cell atlas of the mouse primary motor cortex (Yao et al., 2021), including its spatial organization (Zhang et al., 2021) and comparison across human, marmoset (a new world monkey) and mouse (Bakken et al., 2021). The integrative analysis of seven scRNA-seq and snRNA-seq datasets led to the identification of 116 cell types, counting 59 classes of inhibitory and 31 classes of excitatory neurons, highlighting their large transcriptional diversity. Non-neuronal cells were categorized into 26 clusters, of which eight classes defined the populations of OLs (Table 2). As the BICCN is mostly a neuron-oriented effort, OL heterogeneity receives less attention and many of the interesting findings are waiting to be revealed by the community. The ultimate goal of BICCN is to perform the complete characterization of mouse and human CNS, and therefore another wealth of knowledge is expected in the upcoming years.

The recent developments in spatial transcriptomic technologies have made it possible to correlate OL transcriptional variation to their anatomical location. Floriddia et al. (2020) inspected spatial distribution of three populations of mature OLs in white and gray matter (GM) of murine brain and spinal cord. Specifically, they focused on populations of MOL1, MOL2, and MOL5/6 as defined by Marques et al. (2016), which showed the most distinctive expression profiles. Using a limited number of marker genes, the authors demonstrated different spatial preference and response to spinal cord injury. Further details were provided by Hilscher et al. (2022), who utilized probabilistic cell typing by *in situ* sequencing (pciSeq; Qian et al., 2020), and measured the expression of 124 marker genes of all 12 OL populations as described by Marques et al. (2016). The study focused on murine GM and WM in brain and spinal cord at postnatal, juvenile and young adult age, and revealed age and region related alterations in the composition of OL populations. Together, these two pioneering efforts provided the first hints for the understanding of the functional roles of the OL populations with respect to their anatomical locations.

## Oligodendrocyte heterogeneity in disease

Falcao et al. (2018) represents a landmark study in understanding OL heterogeneity in disease, describing several disease specific OL populations in the spinal cord of mice induced by experimental autoimmune encephalomyelitis (EAE), which is an experimental model of multiple sclerosis (MS). In total, the authors identified 14 clusters, including three populations specific for the controls and five specific for EAE. Notably, even when separated into controls and EAE clusters, all the OLs resembled the clusters from Marques et al. (2016). The analysis of major data trends revealed modules associated with EAE (Table 2), composed of genes related to interferon response, antigen processing and presentation *via* the major histocompatibility complex class I and II (MHC-I and -II) and immune protection, represented by the *Serpina3* gene family. Altogether, the data showed active immunomodulatory function of OLs in EAE, contesting the long-term dogma of their passive role with limited responsiveness to pathogenic stimuli. Extending the study, Jakel et al. (2019) performed an analysis of OLs in human samples with various levels of MS pathology. They identified a cluster of immune *CD74*^+^ OLs (ImOLs), resembling the data from the mouse EAE model. Notably, the recent study by Meijer et al. (2022) showed epigenomic priming of immune genes, further confirming OL immunomodulatory role. Moreover, the positioning of MS susceptibility single-nucleotide polymorphisms (SNPs) within the accessible regulatory regions of genes involved in immune regulation, suggested an altered function of OLs in disease progression.

The initial findings on OL heterogeneity in EAE/MS were soon accompanied by reports from intensive Alzheimer’s disease (AD) research. Focusing on the murine AD model, Zhou et al. (2020) reported *Serpina3n*^+^ *C4b*^+^ reactive OL population (Table 2), specifically enriched in plaque-bearing regions. The gene signature markedly overlapped with EAE-enriched populations of OLs identified earlier by Falcao et al. (2018), suggesting a shared response of OLs in two distinct neurodegenerative models. The identical population of reactive OLs (annotated MOL-DA1) was subsequently confirmed by Lee et al. (2021), who assayed OL heterogeneity in AD models of amyloidosis and tauopathy (Table 2). Moreover, combined tau-amyloid pathology showed more extensive OL response, giving rise to population of OLs with strong Tp53 signaling (MOL-DA2), potentially leading to cell-cycle arrest and apoptosis, however, without any prominent loss of OLs. *In situ* hybridization (ISH) across brain regions confirmed the existence of cell clusters in affected areas and absence in control old animals except aged WM, suggesting age related neurodegenerative changes in this compartment. Notably, both astrocytes and OLs contributed substantially to the overall *C4b* and *Serpina3n* signal. Finally, a recent study of Kenigsbuch et al. (2022) largely confirmed the findings of the aforementioned reports by defining a population of disease-associated oligodendrocytes (DOLs, Table 2), whose gene signature was found in multiple CNS pathologies, including models of MS, AD and aging. Majority of the 26 markers defining DOLs were related to immune response, and regulated by the transcription factor families Stat/Irf, YY1/NF-κB and Sox9, in accordance with the findings of Meijer et al. (2022), who demonstrated a role of Stat1 in the immune OL population. Using human protein homologs of mouse Serpina3n as a key DOLs marker, authors detected SERPINA3 in human AD samples, demonstrating the relevance of the mouse data for human pathology. This, however, contrasts the report of Zhou et al. (2020), who did not detect reactive OLs in human AD samples using snRNA-seq, or Chen et al. (2020), who screened plaque regions for *C4A/C4B* and *SERPINA3* transcripts by *in situ* sequencing (ISS).

Turning attention to human AD samples, OL heterogeneity has been interrogated in several studies investigating multiple brain regions (Del-Aguila et al., 2019; Grubman et al., 2019; Mathys et al., 2019; Lau et al., 2020; Zhou et al., 2020; Gerrits et al., 2021; Leng et al., 2021; Morabito et al., 2021; Sadick et al., 2022). Majority of studies concluded dysregulated OL functions in AD, including changes in differentiation, myelination and metabolic adaptation to neuronal degeneration. OLs have been accented as important players in disease progression, showing sexual dimorphism (Mathys et al., 2019), dysregulation of AD susceptible genes (Grubman et al., 2019), and expression of potential targets for novel AD therapeutics (Morabito et al., 2021). Interestingly, distinctive immune related response observed in murine models of MS and AD were not captured in any of the human studies, except for the very rare *CD74*^+^ OLs (counting for 0.001% of all OLs) identified by Morabito et al. (2021) and a minor cluster of antigen presenting OLs identified by Sadick et al. (2022). Moreover, these cells were not characterized by the expression of neither *C4B* nor *SERPINA3*, which constitute the key markers of reactive OLs, alias DOLs.

The lack of immune OL signature in AD samples was recently scrutinized by Pandey et al. (2022). Using multi-dataset integration, the authors defined three distinct activation states of OLs across the mouse models of AD and MS (MOL-DA1, MOL-DA2, and MOL-IFN), which were characterized by expression of inflammatory-, survival- and interferon-associated genes (Table 2). Follow-up integrative analysis of human OL datasets revealed similar gene signatures in MS patients, but not in AD, indicating a distinct transcriptional response of OLs in human AD pathology. The latest contribution to the understanding of OL heterogeneity in neurodegeneration was provided by Kaya et al. (2022), who studied WM aging in mouse. The authors identified clusters of aging-related *Serpina3n*^+^ *C4b*^+^ OLs (AROs) and interferon-responsive *Stat1*^+^ *B2m*^+^ OLs (IROs) characterized by the expression of type I interferon response genes and MHC-I genes (Table 2). The two populations resembled the inflammatory- and interferon- clusters described by Pandey et al. (2022), but the population presumably involved in the OL survival (MOL-DA2) was missing. Altogether, the data indicates shared, but also distinct response of OLs in different pathologies that requests further investigation.

To date, single-cell and single-nucleus transcriptomics have been applied to most neurodegenerative and neuropsychiatric disorders, e.g., amyotrophic lateral sclerosis (Pineda et al., 2021), Parkinson’s disease (Smajic et al., 2022), schizophrenia (Ruzicka et al., 2020), major depressive disorders (Nagy et al., 2020), and autism (Velmeshev et al., 2019). Unfortunately, the analysis of OLs has often not been of primary interest and therefore not explored in detail. Other CNS disorders with a recently discovered role of OLs in its disease etiology, e.g., epilepsy (Knowles et al., 2022), are still awaiting in-depth single-cell transcriptomic characterization. Attention is also required for the OLs in the acute CNS injuries, where their role is largely unexplored.

## Conclusion

We provide a comprehensive overview of key studies defining the current spectrum of OL heterogeneity in health and disease. We document a first standardized OL nomenclature in murine healthy CNS and strong indication of distinct reactive immune OL gene signature present in multiple models of CNS pathologies. OL heterogeneity in human is less defined and there are distinct transcriptional OL phenotypes present in MS and AD patients. Future studies are needed to establish a robust nomenclature of human OLs and characterize the full spectrum of OL activation states in other CNS disorders.

## Author contributions

LV, ZM, and DZ drafted the manuscript. RK, SB, PA, MK, and MA participated in subsequent review and editing process. All authors have read and agreed to the published version of the manuscript.
